# Supplementary material for: Identification of photoperiod-induced specific miRNAs in the adrenal glands of Sunite sheep (Ovis aries)
Source: Front Vet Sci. 2022 Jul 22;9:888207. doi: 10.3389/fvets.2022.888207 (PMC9354845; doi:10.3389/fvets.2022.888207)
Supplement: Supplementary file 1 [file Data_Sheet_1.ZIP › Supplementary file/miRDeep2 file.zip!/miRDeep2 file/Know_Structure/SP42A1/oar-mir-29a.pdf]

miRBase precursor : oar-mir-29a
Total read count : 97948
oar-miR-29a read count : 97930
remaining reads : 18

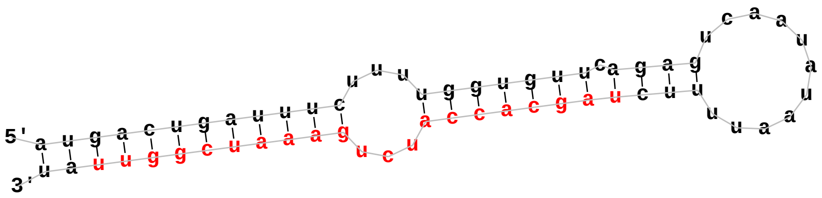

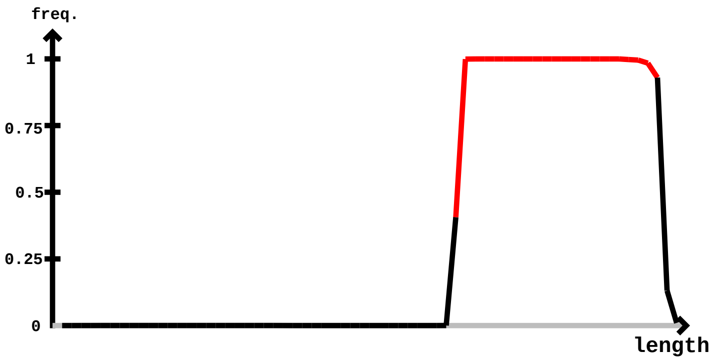

oar-miR-29a

```
5'- augacugauuucuuuugguguucagagucaauauaauuuucuagcaccaucugaaaucgguuau   -3'    exp
    ((((((((((((...(((((((.((((...........)))))))))))...))))))))))))    reads      mm         sample
    augacugauuucuuuugguguu..........................................    1          0          seq
    .ugacugauuucuuuugguguu..........................................    2          0          seq
    .ugacugauuucuuuugguguuc.........................................    5          0          seq
    ...acugauuucuuuugguguu..........................................    1          0          seq
    ...acugauuuUuuuugguguuc.........................................    1          1          seq
    ...acugauuucuuuugguguuc.........................................    8          0          seq
    .......................................ucuagcaccaucugaaaucgg....    3          0          seq
    .......................................ucuagcaccaucugaaaucggu...    8          0          seq
    .......................................ucuagcaccaucugaaaucgguu..    4          0          seq
    .......................................ucuagcaccauUugaaaucgguu..    1          1          seq
    ........................................cuagcaccaucugaaau.......    5          0          seq
    ........................................cuagcaccaucugaaGu.......    1          1          seq
    ........................................cuagcaccaucugaaauA......    4          1          seq
    ........................................cuagcaccaucugaaauc......    36         0          seq
    ........................................cuagcaccaucugaaaucg.....    47         0          seq
    ........................................cuagcaccaucugaaaucgg....    181        0          seq
    ........................................cuagcaccauUugaaaucggu...    24         1          seq
    ........................................cuagcaccaucugaaaucggu...    1514       0          seq
    ........................................cuagcaccaucugaaaucgguu..    37049      0          seq
    ........................................cuagcaccauUugaaaucgguu..    111        1          seq
    ........................................cuagcaccauUugaaaucgguua.    1          1          seq
    ........................................cuagcaccaucugaaaucgguua.    747        0          seq
    ........................................cuagcaccaucugaaaucgguuau    73         0          seq
    .........................................uagcaccaucugaGauc......    1          1          seq
    .........................................uagcaccaucugaaauU......    12         1          seq
    .........................................uagcUccaucugaaauc......    1          1          seq
    .........................................uagcaccaucugaaauA......    7          1          seq
    .........................................uagcaccaucugaaauc......    156        0          seq
    .........................................uagcaccauUugaaaucg.....    9          1          seq
    .........................................uagcaccaucugaaaucg.....    150        0          seq
    .........................................uagcaccaucugaaaucgg....    853        0          seq
    .........................................uagcaccauUugaaaucgg....    87         1          seq
    .........................................uagcaccauUugaaaucggu...    400        1          seq
    .........................................uagcaccaucugaaaucggu...    3354       0          seq
    .........................................uagcaccauUugaaaucgguu..    1733       1          seq
```

oar-miR-29a

augacugauuucuuuugguguucagagucaauauaauuuuc**uagcaccaucugaaaucgguu**au			
....uagcaccaucugaaaucgguu..	39310	0	seq
....uagcaccaucugaaaucgguua.	10588	0	seq
....uagcaccauUugaaaucgguua.	504	1	seq
....uagcaccauUugaaaucgguuau	43	1	seq
....uagcaccaucugaaaucgguuau	854	0	seq
....agcaccaucugaaaucggu...	1	0	seq
....agcaccaucugaaaucgguu..	33	0	seq
....agcaccaucugaaaucgguua.	12	0	seq
....agcaccauUugaaaucgguua.	1	1	seq
....gcaccaucugaaaucgguu..	7	0	seq
....gcaccaucugaaaucgguua.	2	0	seq
....caccauUugaaaucgguua.	1	1	seq
....accaucugaaaucgguu..	1	0	seq
....ccaucugaaaucgguua.	1	0	seq
